# Combined Modification of Fiber Materials by Enzymes and Metal Nanoparticles for Chemical and Biological Protection

**DOI:** 10.3390/ijms23031359

**Published:** 2022-01-25

**Authors:** Ilya Lyagin, Nikolay Stepanov, George Frolov, Elena Efremenko

**Affiliations:** 1 Faculty of Chemistry, Lomonosov Moscow State University, Lenin Hills 1/3, 119991 Moscow, Russia; lyagin@mail.ru (I.L.); na.stepanov@gmail.com (N.S.); 2 N.M. Emanuel Institute of Biochemical Physics RAS, Kosygin str. 4, 119334 Moscow, Russia; 3 Department of Physical Chemistry, National University of Science and Technology “MISIS”, Leninsky ave. 4, 119049 Moscow, Russia; georgifroloff@yandex.ru

**Keywords:** fiber material, nanoparticle, antibiotic, enzyme, destruction, organophosphorus compound, chemical and biological protection, toxin

## Abstract

To obtain fiber materials with pronounced chemical-biological protection, metal (Zn or Ta) nanoparticles were jointly applied with polyelectrolyte complexes of enzymes and polypeptides being their stabilizers. Computer modeling revealed the preferences between certain polyelectrolyte partners for *N*-acyl-homoserine lactone acylase and hexahistidine-tagged organophosphorus hydrolase (His_6_-OPH) possessing the quorum quenching (QQ) behavior with bacterial cells. The combinations of metal nanoparticles and enzymes appeared to function better as compared to the combinations of the same QQ-enzymes with antibiotics (polymyxins), making it possible to decrease the applied quantities by orders of magnitude while giving the same effect. The elimination of Gram-positive and Gram-negative bacterial cells from doubly modified fiber materials notably increased (up to 2.9-fold), whereas His_6_-OPH retained its hydrolytic activity in reaction with organophosphorus compounds (up to 74% of initially applied activity). Materials with the certain enzyme and Zn nanoparticles were more efficient against *Bacillus subtilis* cells (up to 2.1-fold), and Ta nanoparticles acted preferentially against *Escherichia coli* (up to 1.5-fold). Some materials were proved to be more suitable for combined modification by metal nanoparticles and His_6_-OPH complexes as antimicrobial protectants.

## 1. Introduction

Currently, chemical and biological safety still plays a huge role in human life in various military and peacetime operations, including chemical attacks, man-made disasters caused by the leakage of chemicals and biological agents, the use of pesticides in agriculture, contact with pathogenic microorganisms during the storage of agricultural products, etc. [1,2,3]. Most materials that can provide both chemical and biological defense to consumers are highly specialized, and their use is mostly limited to military and other special agencies. Thus, many people encountering dangerous agents on a daily basis are commonly unprotected.

The general approach to countering chemical and biological hazards is still isolation from them [4]. Meanwhile, there is a tremendous progress in the development of novel materials, especially nanomaterials [5], allowing not only prevention of their direct impact on humans, but also the active elimination of such biological pathogens and toxic chemical compounds [6]. Universal materials eliminating both chemical and biological hazards seem to be the most promising for application. In this vein, several approaches have been proposed, using: (i) inorganic metal nanoparticles (NPs), e.g., ZnO [7] and other metal oxides [8]; (ii) nanocomposites, e.g., Ag@Al_2_O_3_ [9] and Ag@ZnTiO_3_ [10]; (iii) enzymatic biocatalysts, e.g., combinations of horseradish peroxidase and glucose oxidase with chemically modified carriers [11]; etc. Such materials can be photoresponsive (i.e., have photocatalytic activity) [12,13], although there are some doubts about the applicability of such methods in general practice.

It is reasonable and may be interesting to combine multipurpose active substances into a single multifunctional material, for example, by its simultaneous modification with metal nanoparticles and enzyme(s). Previously, a simple method was applied to typical fibers to achieve their modification by metal NPs [14], and the best bactericidal activity was observed with Ta and Zn. On the other hand, many other antimicrobials (antibiotics, antimicrobial peptides, hydrolytic enzymes) can also be applied with fiber materials for the bacterial cell disruption and material self-sterilization. To improve their action, combination of antimicrobials with enzymes, hydrolyzing the main molecules responsible for the developing of Quorum sensing between cells of microorganisms, and thereby increasing their resistance to antimicrobials, is useful. For example, enzyme hexahistidine-tagged organophosphorus hydrolase (His_6_-OPH) is known to improve antibacterial activity of selected antimicrobial peptides [15,16] and can catalyze hydrolysis of various neurotoxic organophosphorus compounds (OPCs) [17,18]. Moreover, complexes of His_6_-OPH with polyelectrolytes are even better in both functionalities [19,20]. At the same time, the polyelectrolyte complexes of the enzyme notably stabilize the enzymatic activity and provide the long-term use of such improved antimicrobials.

Thus, the aim of the current work was to combine His_6_-OPH and bactericide fiber materials modified by metal NPs to obtain multifunctional protective materials (Scheme S1). Since *N*-acyl-homoserine lactone acylase PvdQ (PvdQ) and/or penicillin G acylase (PGA) have previously shown positive results in improving the suppression of bacterial quorum sensing and the antimicrobial action of conventional antibacterials [16], these enzymes were used for comparison in a similar methodology. The action of the developed samples of fiber materials with dual modifications was tested using the Gram-positive bacteria *Bacillus subtilis* and Gram-negative bacteria *Escherichia coli* cells, whereas the hydrolytic activity of the His_6_-OPH within materials was controlled in reaction with organophosphorus pesticide paraoxon. In this work, various polypeptides, namely polyglutamic (PLE_50_) and polyaspartic (PLD_50_) acids, their PEGylated derivatives (PEG-PLE_50_, PEG-PLD_50_, PLE_50_-PEG-PLE_50_) and succinylated gelatin, which have previously been investigated for His_6_-OPH [20], were estimated as partners for the PGA in enzyme–polyelectrolyte complexes for the first time. The known polypeptide antibiotics (polymyxin B and polymyxin E), previously used as a component of polyelectrolyte complexes for His_6_-OPH and PGA and which demonstrated good efficiency with these enzymes in bacterial cellulose [16], were applied in this investigation for combinations with enzymes and for modifications of fiber materials. Typical semisynthetic fiber materials that are widely commercially available and composed of a blend of viscose and polyester were used in the work.

## 2. Results

### 2.1. Modeling of Enzyme–Polyelectrolyte Complexes and Their Activity within Bacterial Cellulose

**Figure 1:**
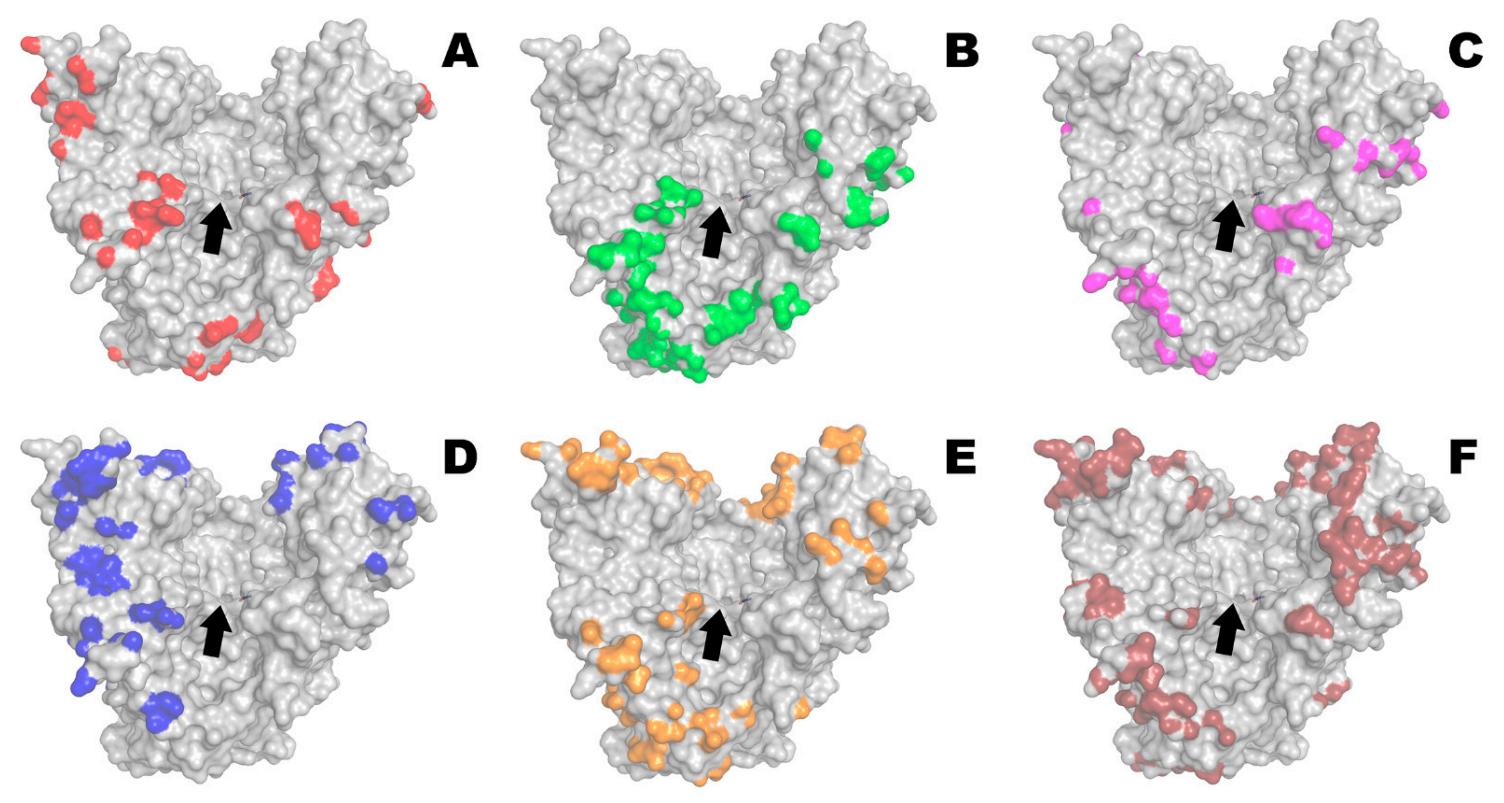
Domains of PvdQ (PDB 4m1j) covered by PLE_50_ (**A**), PLD_50_ (**B**), PEG-PLE_50_ (**C**), PEG-PLD_50_ (**D**), PLE_50_-PEG-PLE_50_ (**E**), and succinylated gelatin (gelofusine) (**F**), and thus capable of interacting with them within enzyme–polyelectrolyte complexes. The entrance into the active site is highlighted by the arrow; it localizes in the hollow of the V-shaped protein molecule, being slightly tilted forward in the figure for illustration purposes.

Possible interactions of PvdQ with different polymers that have previously been shown to be good (PLE_50_, PLD_50_, PEG-PLE_50_, PEG-PLD_50_, PLE_50_-PEG-PLE_50_) or poor (succinylated gelatin) partners for His_6_-OPH [20] were evaluated at neutral pH 7.5 (i.e., close to the optimum pH of the enzyme) (Figure 1).

**Figure 2:**
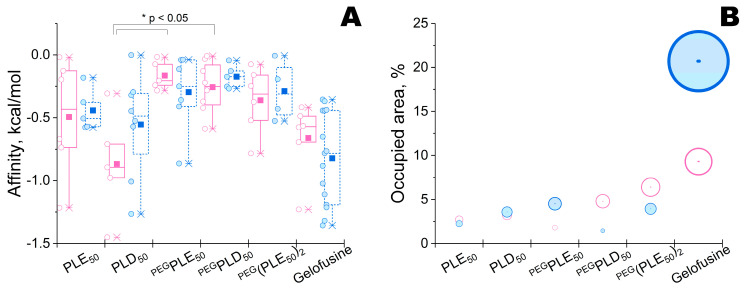
Affinity (**A**) of PLE_50_, PLD_50_, PEG-PLE_50_, PEG-PLD_50_, PLE_50_-PEG-PLE_50_ and succinylated gelatin (gelofusine), and the relative area (**B**) which they occupy on the surface of PvdQ (○) or His_6_-OPH (●) at pH 7.5. Designations: mean value (■), 1st and 99th percentile (×); interquartile ranges are bordered, median values are presented by horizontal lines, and data points are aligned to the left. The diameters of bubbles in (**B**) are proportional to the values of occupied areas with a scaling factor of 6 for illustration purpose. Data for His_6_-OPH correspond topreviously obtained [20].

The lowest and highest binding energies (i.e., affinity) were estimated for PLD_50_ and PEG-PLE_50_, respectively (Figure 2). Though there was a statistically significant difference in the means of affinity (according to one-way ANOVA, *p* = 0.007), only groups PLD_50_ vs. PEG-PLE_50_ and PLD_50_ vs. PEG-PLD_50_ differed enough (according to Holm-Sidak pairwise multiple comparison method, *p* = 0.017 and *p* = 0.022, respectively), while the other groups had no statistically significant difference.

The minimal and maximal areas were determined for PEG-PLE_50_ and succinylated gelatine (gelofusine), respectively (Figure 2). Cumulatively, PLE_50_ was selected as a trade-off between the strength of complexing with enzyme and the possible negative influence on enzyme activity owing to occupation of enzymatic active site.

As compared to His_6_-OPH, there was a variability in affinity (e.g., in the case of PLD_50_) or in occupied area (e.g., in most cases, excepting PLE_50_ and PLD_50_). Thus, PLE_50_ could be a universal partner for both His_6_-OPH and PvdQ, and could possibly be approximated for other enzymes. Interestingly, only succinylated gelatin (gelofusine) appeared to be an absolutely poor partner for both enzymes.

**Figure 3:**
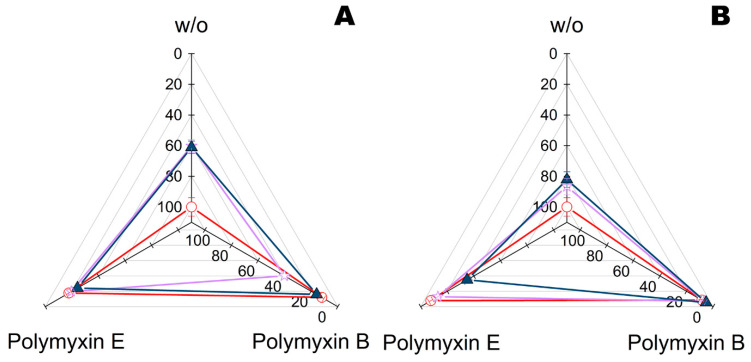
Residual intracellular ATP quantity (%) in *B. subtilis* (**A**) and *E. coli* bacterial cells (**B**) exposed for 24 h to bacterial cellulose treated with polymyxin B or polymyxin E, enzymes (His_6_-OPH or PGA), or their combinations. ATP of bacterial cells similarly exposed on the same bacterial cellulose with treatment by buffer (i.e., controls) was taken as 100%. All solutions were prepared in a PBS buffer (pH 7.4), and enzymes were additionally stabilized in complexes with PLE_50_. Designation: ○—control without enzyme, ✩—with PGA, ▲—with His_6_-OPH, w/o—without polymyxins.

Furthermore, complexes of PLE_50_ with PGA or His_6_-OPH and with additions of polymyxin B or polymyxin E (colistin) were issued towards Gram-positive bacteria *Bacillus subtilis* and Gram-negative bacteria *Escherichia coli* according to a previously developed method using bacterial cellulose [16] (Figure 3).

At neutral values of pH, the complexing of enzymes with both PLE_50_ and polymyxins had some effect on the antibacterial activity of the latter. These effects were minimal for His_6_-OPH vs. *B. subtilis* and for PGA vs. *E. coli*. Combined preparations based on His_6_-OPH showed more constant characteristics with polymyxin B, while PGA preferred combination with polymyxin E.

### 2.2. Combination of Metal Nanoparticles with Enzymes within Fiber Materials

**Figure 4:**
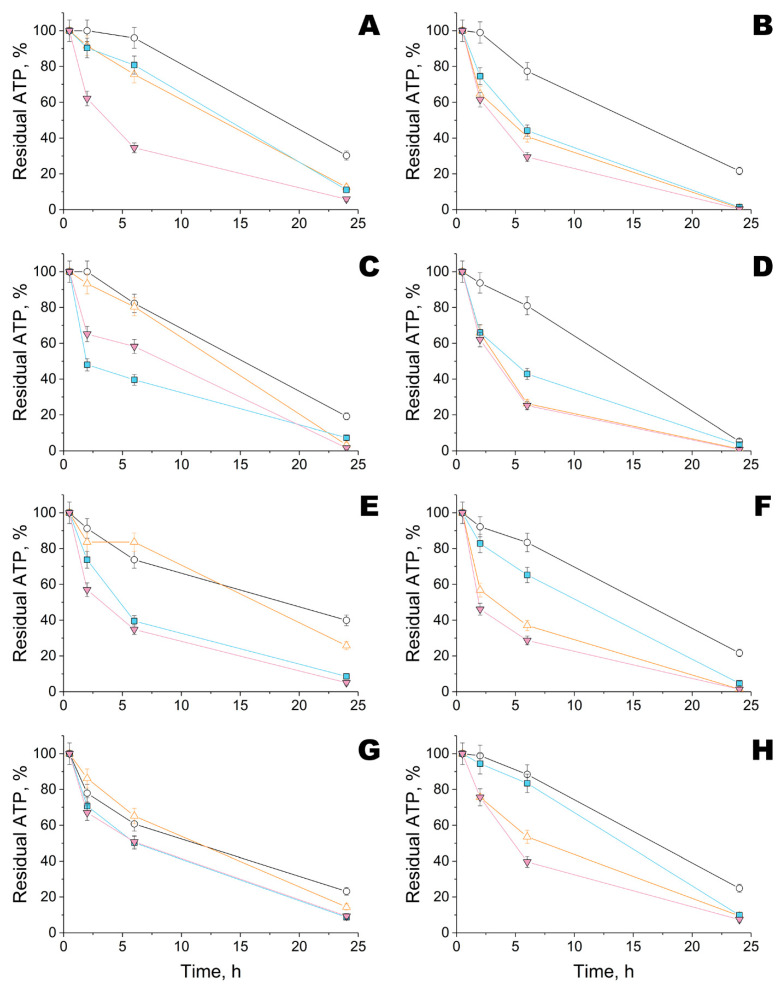
Kinetics of residual intracellular ATP quantity in *B. subtilis* (**A**,**C**,**E**,**G**) and *E. coli* bacterial cells (**B**,**D**,**F**,**H**) during their exposure on different fiber materials #1 (**A**,**B**), #2 (**C**,**D**), #3 (**E**,**F**) and #4 (**G**,**H**) treated with Ta NPs, enzyme His_6_-OPH, or their combinations. ATP quantity in bacterial cells at 0.5 h was taken as 100%. The His_6_-OPH was prepared as a complex with PEG-PLE_50_ in a Na-carbonate buffer (pH 10.5). Designation: ○—control, ■—Ta NPs, ∆—His_6_-OPH, ▼—Ta NPs with His_6_-OPH.

Initially, the kinetics of bacterial elimination was investigated for materials modified by Ta NPs and complexes of His_6_-OPH with PEG-PLE_50_ obtained at pH 10.5 (i.e., pH-optimum of the enzyme) (Figure 4). Such complexes of His_6_-OPH with PEG-PLE_50_ have previously been shown to provide maximum stabilization under harsher conditions [21].

Viable cells gradually decreased over time in all variants. However, Ta NPs alone or in combination with His_6_-OPH significantly increased the rate of cell death. The highest degree of cell death at 24 h exposure on materials #1, #2 and #3 was in the case of combination with His_6_-OPH, irrespective of the metal NPs used (Ta or Zn) (Figure S1). The highest efficiency towards both microorganisms was revealed for combination with Ta NPs on materials #1, #2 and #3.

**Figure 5:**
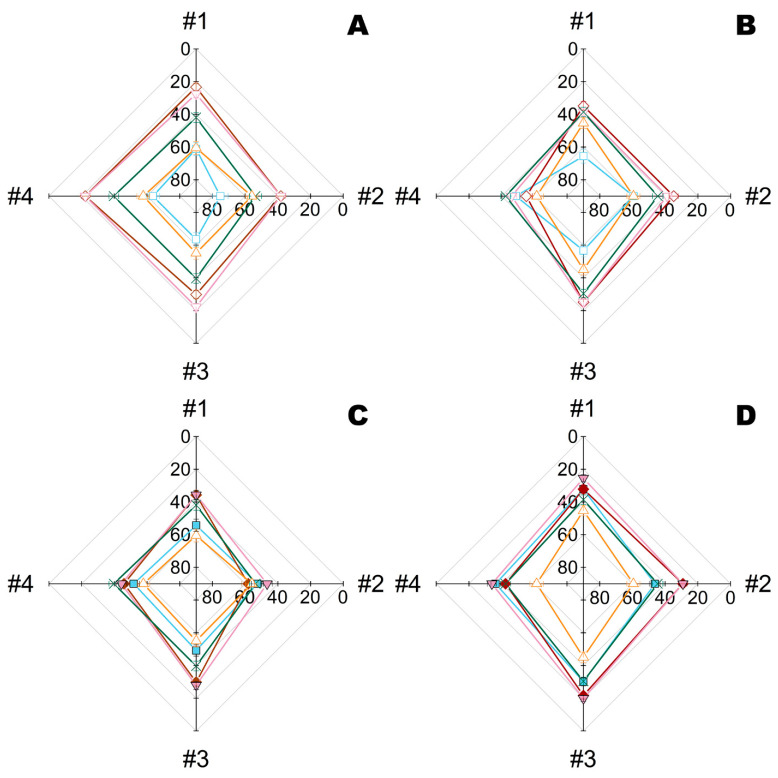
Residual intracellular ATP quantity (%) in *B. subtilis* (**A**,**C**) and *E. coli* bacterial cells (**B**,**D**) exposed for 24 h on multiple fiber materials (#1, #2, #3, and #4) treated with Zn (**A**,**B**) or Ta NPs (**C**,**D**), enzymes (His_6_-OPH or PGA), or their combinations. ATP of bacterial cells similarly exposed on the same materials with treatment by buffer (i.e., controls) was taken as 100%. All solutions were prepared in a Na-carbonate buffer (pH 9.5) and enzymes were additionally stabilized in complexes with PLE_50_. Designation: □—Zn NPs, ×—PGA, ∆—His_6_-OPH, ◇—Zn NPs with, ▽—Zn NPs with His_6_-OPH, ■—Ta NPs, ◆—Ta NPs with PGA, ▼—Ta NPs with His_6_-OPH.

Further both PGA and His_6_-OPH complexes were similarly obtained and tested under the same conditions at pH 9.5 (i.e., intermediate value between the pH-optima of these enzymes) (Figure 5). As previously, combinations with enzymes appeared to be preferable for metal NPs alone, while both enzymes in combination were almost undistinguishable between each other.

Interestingly, combinations with metal NPs seem to have variable specificity towards microorganisms, and as a result, combinations with Zn and Ta were most effective towards *B. subtilis* and *E. coli* cells, respectively. The antimicrobial activity of materials #1, #3 and #4 appeared to be slightly more pronounced towards *B. subtilis*, and materials #1, #2 and #3 were more effective towards *E. coli*.

### 2.3. Combination of Polymyxins with Enzymes within Fiber Materials

**Figure 6:**
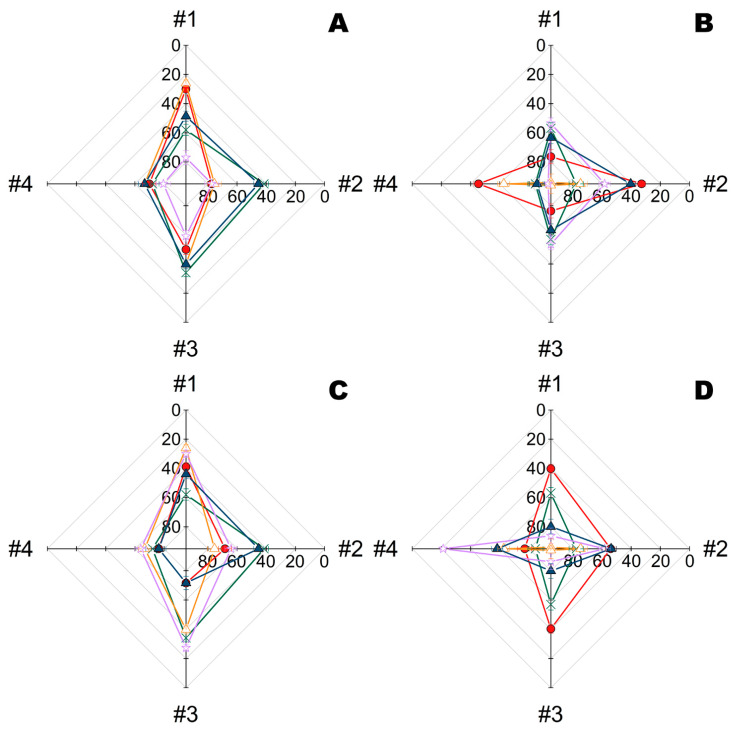
Residual intracellular ATP quantity (%) in *B. subtilis* (**A**,**C**) and *E. coli* bacterial cells (**B**,**D**) exposed for 24 h on multiple fiber materials (#1, #2, #3, and #4) treated with polymyxin B (**A**,**B**) or polymyxin E (**C**,**D**), enzymes (His_6_-OPH or PGA), or their combinations. ATP of bacterial cells similarly exposed on the same materials with treatment by buffer (i.e., controls) was taken as 100%. All solutions were prepared in a PBS buffer (pH 7.4). Designation: ●—polymyxin, ×—PGA, ∆—His_6_-OPH, ✩ –PGA with polymyxin, ▲—His_6_-OPH with polymyxin.

**Figure 7:**
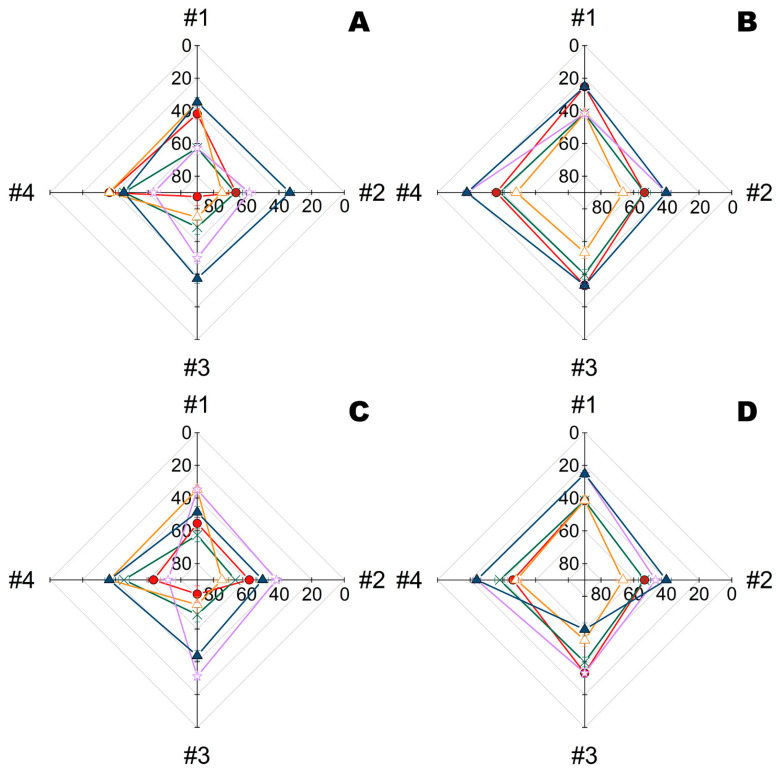
Residual intracellular ATP quantity (%) of *B. subtilis* (**A**,**C**) and *E. coli* bacterial cells (**B**,**D**) exposed for 24 h on multiple fiber materials (#1, #2, #3, and #4) treated with polymyxin B (**A**,**B**) or polymyxin E (**C**,**D**), enzymes (His_6_-OPH or PGA), or their combinations. ATP of bacterial cells similarly exposed on the same materials with treatment by buffer (i.e., controls) was taken as 100%. All solutions were prepared in a Na-carbonate buffer (pH 9.5) and enzymes were additionally stabilized in complexes with PLE_50_. Designation: ●—polymyxin, ×—PGA, ∆—His_6_-OPH, ✩ –PGA with polymyxin, ▲—His_6_-OPH with polymyxin.

To reveal the applicability of the procedure, conventional antibiotics were applied in combination with enzymes to fiber materials under the same protocol (Figure 6 and Figure 7).

As opposed to bacterial cellulose, the structure of fiber material significantly influenced the efficiency of the polymyxins and their combinations with enzymes. The highest synergic effect at pH 7.4 towards *B. subtilis* (Figure 6) was found in combination of polymyxin E with PGA loaded onto materials #1 and #3, while towards *E. coli* it was for the same enzyme on material #4. The His_6_-OPH was in the middle, though it slightly preferred material #2 and polymyxin B.

The picture changed drastically when pH was increased to 9.5 (Figure 7). The combination of polymyxin B with His_6_-OPH became the absolute leader towards both *B. subtilis* and *E. coli*, irrespective of the material used. PGA showed comparable efficiency only with polymyxin E on materials #1, #2 and #3 towards *B. subtilis*, with polymyxin B on materials #2, #3 and #4 towards *E. coli*, or with polymyxin E on materials #1, #2 and #4 towards *E. coli*.

### 2.4. Activity of His_6_-OPH within Modified Fiber Materials

**Figure 8:**
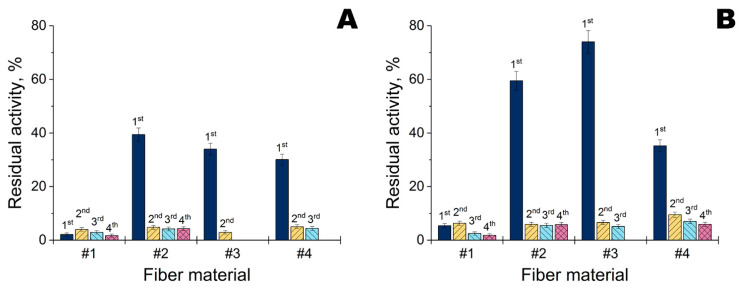
Residual activity of His_6_-OPH loaded onto multiple fiber materials (#1, #2, #3, and #4) treated with Zn (**A**) or Ta NPs (**B**) during repeated application. Initial loaded activity of His_6_-OPH (1 U) was taken as 100%. The His_6_-OPH was prepared as a complex with PEG-PLE_50_ in a Na-carbonate buffer (pH 10.5). Absent columns indicate that residual activity was equal to or less than limit of determination (0.01 U).

Activity of His_6_-OPH within modified fiber materials was investigated with a paraoxon as substrate at pH 10.5 (Figure 8). A lot of enzyme activity was retained within materials #2, #3 and #4. It was maximal with Ta NPs, and especially on material #3. However, enzyme activity was dramatically reduced on the 2nd and subsequent application cycles. The best retention of activity was observed during the 2nd and subsequent repeated uses of material #2, irrespective of the applied metal NPs.

## 3. Discussion

The formation of enzyme–polyelectolyte complexes makes it possible to significantly stabilize enzymes in harsh environmental conditions [17,19,20,21]. Since protective materials are supposed to be applied under such conditions, the enzymes within them have to be stabilized somehow. Though such stabilized preparations of His_6_-OPH have been developed already, complexes for an alternative enzyme—PGA—were designed in the current work for the first time. Despite this not being the main purpose of the work, complexes of PGA with PLE_50_ appeared to be quite useful and could be interesting. Namely, the lowest affinity for PLD_50_ could result in too strong binding of polymer to enzyme, and thus in losses of activity due to hindrances to structural mobility. On the other hand, the highest affinities being observed for PEGylated polypeptides, especially PEG-PLE_50_, are likely to lead to unstable complexes, and thus to lower enzyme stabilization. Succinylated gelatin (gelofusine) had comparable affinity with PLE_50_ but also occupied too much area on the enzyme surface, which could also result in activity loss [20]. Thus, PLE_50_ is a consensus polymer and also applicable with His_6_-OPH.

At present, there are several major paradigms of formation of stable/resistant microbial communities and their destruction by action of organic and inorganic active compounds [22]. In this continuum, enzymes (like PGA or His_6_-OPH) are supposed to intercept and destroy simple molecular messengers used by bacteria in their interaction in the so-called Quorum Sensing (QS) state [23]. Namely, both PGA and His_6_-OPH hydrolyze N-acyl homoserine lactones (AHLs) of some Gram-negative bacteria. Both Gram-positive *B. subtilis* and Gram-negative *E. coli* cells are not known to use any AHLs in their QS. This raises a question as to how it is possible that PGA and His_6_-OPH have any positive effect in the current experimental design with antibacterials of two different modalities (i.e., NPs and polymyxins). Hypothetically, for these enzymes there can be other metabolic targets that play important role within cells. Otherwise, bacteria may shift their metabolome during QS and the subsequent colonization of the fiber materials and bacterial cellulose, with the enzymes acting on metabolites other than AHLs. Yet another option, proposed previously [15], is that enzymes act as a ‘shuttle’ for antibacterials to penetrate into bacterial cells. Currently, it is not possible to make a certain conclusion as to the mechanism of the observed effects. However, further studies are required to elucidate these results.

From a practical standpoint, combinations of metal NPs with enzymes seem preferable to combinations of the same enzymes with polymyxins. Firstly, the quantities of NPs required to achieve a similar effect were 5.6 and 21.7 times lower (for Zn and Ta NPs, respectively). For example, less than 5 mg of Ta NPs was applied per 1 m^2^. Secondly, it looks like these NPs did not show any preferences for an enzyme to combine with. Thirdly, the antibiotics used in this work are for medical application, and thus their ‘improper’ usage could intensify development of resistance to these antibiotics in microorganisms.

An *EC*_50_ dosage applied to materials in combination with enzymes resulted in an improvement up to *EC*_80_ for most of the fibers. However, little individual specificity of the combined modification to different bacterial cells was revealed, and materials based on Zn and Ta NPs seemed to be slightly preferable for eliminating *B. subtilis* and *E. coli* cells, respectively.

Currently, it is not possible to directly compare the obtained materials with other examples known from the literature, due to several reasons. Firstly, most authors have used the agar plate antibacterial test (i.e., leaching of nanomaterials) or, much more rarely, the broth test (i.e., immersion of fibers into medium with subsequent leaching of nanomaterials). They are both difficult to apply for the testing of personal protective equipment. Secondly, the content of nanomaterials (if there is any) is larger by orders of magnitude in comparison to the current work, for example, it was at least 200 mg/m^2^ ZnO NPs in [7]; up to 14 g/m^2^ Ag and up to 69 g/m^2^ ZnTiO_3_ NPs in [10]; 11.5 wt.% Ag@TiO2 nanocomposite in [12]; or even cannot be estimated without additional data, as in the case of [9,13].

The fiber materials which were applied as a base also varied to a great extent: nylon 6 [7]; poly(vinylidene difluoride) [9]; multilayer composite (mostly unspecified, excepting layers of polyethylene terephthalate and of activated carbon) [10]; polyurethane [12]; and activated carbon fibers [13]. Additional dimethylacrylamide-methacrylate copolymer was revealed when investigating an ‘enzyme only’ material [11]. Proper selection of the base material was shown in the current work to influence antibacterial and enzyme activity significantly. In summary, materials #2 and #3, both containing polyester (more probably polyethylene terephthalate), appear to be the most suitable for combined modification with metal NPs and His_6_-OPH complexes. Although material #1 showed good antibacterial activity with both NPs and antibiotics, and had similar composition as #3 but a 1.7-fold lower surface density, it exhibited poor retention of His_6_-OPH complexes (Figure 8).

Despite positive results in genere, there are still some problems. Namely, His_6_-OPH seems to be actively eluted from all fiber materials (Figure 8). This process is highly likely to occur simultaneously with elution of metal NPs. In this work, they were simply adsorbed onto the fiber materials, and could be easily desorbed. To prevent such excessive losses, preliminary chemical and/or physical modification of such fibers could be helpful.

## 4. Materials and Methods

### 4.1. Materials

Polymyxin B, polymyxin E (=colistin), penicillin G acylase (PGA) from *E.coli* and paraoxon (diethyl 4-nitrophenyl phosphate) were purchased from Sigma-Aldrich (Darmstadt, Germany). Polyglutamic acid (PLE_50_) and pegylated PLE_50_ (PEG-PLE_50_) were obtained from Alamanda Polymers (Huntsville, AL, USA). Recombinant *E. coli* strain SG13009[pREP4] (Qiagen, Hilden, Germany) transformed by a plasmid encoding His_6_-OPH was used for the production of His_6_-OPH by a patented method and, further, the enzyme was purified by a published procedure [24]. According to methods described earlier [25], His_6_-OPH concentration was determined by Bradford assay with Coomassie Brilliant Blue G-250 (Sigma-Aldrich); protein purity was analyzed by sodium dodecyl sulfate polyacrylamide gel electrophoresis in a 12% polyacrylamide gel using a Mini-PROTEAN II cell (Bio-Rad, Hercules, CA, USA) followed by Coomassie Brilliant Blue R-250 (Sigma-Aldrich) staining, and was ca. 98 ± 0.5%. Enzyme–polyelectrolyte complexes were obtained by previously published method [20,21] by simple mixing of an enzyme and polymer solution at molar ratio 1:1.

Bacterial cellulose was produced with *Komagataeibacter xylinum* B-12429 cells, as previously described for a medium with fructose [26,27], then dried at room temperature overnight under sterile conditions and cut to samples of 1 × 1 cm before further use.

The fibrous materials were purchased locally and used as is. Materials #1 and #3 were nonwoven and consisted of 70% viscose and 30% polyester while differing in thickness. Material #2 had multilayer structure with activated carbon layer between polyester nonwoven fabrics. Material #4 was investigated previously [14], it contained 30% cotton and 70% meta polyaramide fiber fabric and was covered by poly(vinylidene difluoride)-*co*-poly(tetrafluoroethylene) membrane. The water vapor transmission rate through materials #1–#4 over 24 h, measured according to ISO 2528:2017, was 367, 47, 300 and 480 g/m^2^, respectively. The mass per area of materials #1–#4, measured according to ISO 3801:1977, was 32.4 ± 4.5, 426 ± 24, 54.7 ± 4.1 and 343 ± 26 g/m^2^, respectively. These materials were cut to samples of 1 × 1 cm before further use.

### 4.2. Preparation and Characterization of Metal Nanoparticles

Metal NPs were obtained in a plasma electric arc discharge as published previously [14,28] using ethanol as a solvent. They were characterized by size using a transmission electron microscope LEO 912 AB OMEGA (Carl Zeiss, Oberkochen, Germany) with energy filter and Keller system; by size distribution and ζ-potential using dynamic light scattering on a Zetasizer Nano ZS (Malvern Instruments, Worcestershire, UK); by qualitative elemental composition using an atomic-emission spectrometer iCAP 6300 Radial View (Thermo Fisher Scientific Inc., Waltham, MA, USA) with inductively coupled plasma.

### 4.3. Preparation and Antibacterial Activity of Modified Bacterial Cellulose (BC)

All tested BC samples loaded by antimicrobial agents and hydrolytic enzymes were prepared by the same general procedure [16] with minor modifications. To wit, 10 μL of 10 g/L antimicrobial agent and 5 μL of 2.5 g/L PGA (or 1 g/L His_6_-OPH) complexed with PLE_50_ (at molar ratio 1:1) in a PBS buffer (pH 7.4) were sequentially applied to samples of bacterial cellulose (1 cm × 1 cm) and dried for 20–22 h at +8 °C under sterile conditions. Then, antibacterial activity was analyzed according to the previously published procedure [14] with cells of the G(−) bacterium *E. coli* DH5α (Thermo Fisher Scientific, Waltham, MA, USA), and the G(+) bacterium *B. subtilis* B-522 (All-Russian Collection of Microorganisms, Russia). Cells were aerobically cultivated in Luria–Bertani (LB) culture medium on a thermostatically controlled Adolf Kuhner AG shaker (Basel, Switzerland) at 37 °C and agitation of 150 rpm. Cell growth was monitored with an Agilent UV-8453 spectrophotometer (Agilent Technology, Waldbronn, Germany) at 540 nm. Bacterial cells were grown for 18–20 h, and then separated from the culture broth by centrifugation at 8000× *g* for 10 min (Avanti J25, Beckman, Brea, CA, USA). Cell biomass was suspended in a sterile 0.9% NaCl solution and 20 μL of suspension with cells concentration of (2 ± 0.5) × 10^6^ cells/mL was loaded onto the BC samples. After 24 h of exposure, samples were placed in 1 mL of DMSO and gently stirred. Following 3 h of extraction, the residual concentration of ATP in the extract was determined using a standard luciferin–luciferase ATP reagent (Lyumtek Ltd., Moscow, Russia) by a known protocol [29,30]. BC samples containing only individual antimicrobial agents (i.e., polymyxin B or colistin) without enzymes were used as positive controls, while BC samples similarly treated with PBS buffer only were used as negative controls. The intensity of bioluminescence was measured using a Microluminometer 3560 (New Horizons Diagnostic, Arbutus, MD, USA). The calibration curves for the determination of cell concentrations were plotted, where the concentration of ATP was used as a function of the concentration of colony-forming units (CFU), calculated by a traditional microbiological method, using agar-containing media.

The effective concentrations of antimicrobials leading to a 50% decrease in the amount of living cells were assumed as values of *EC*_50_. The experiments were undertaken no less than in triplicate.

### 4.4. Preparation and Antibacterial Activity of Modified Fibrous Materials

Metal NPs were deposited onto fibrous materials as described previously [14], with minor modifications. Briefly, 25 μL of 18.3 mg/L Ta (or 71.7 mg/L Zn) NPs in ethanol were dropwise loaded per sample (1 × 1 cm) and then dried overnight at a room temperature within Petri dishes. Then, 5 μL of 0.3 g/L His_6_-OPH (or 0.5 g/L PGA) complexed with PLE_50_ in a PBS buffer (pH 7.4) or Na-carbonate buffer (pH 9.5) were applied and dried for 20–22 h at +8 °C under sterile conditions. After that, antibacterial activity was determined as described above for BC. Samples treated with both NPs and buffers were used as positive controls, while samples similarly treated only with buffers were used as negative controls.

Analogously, conventional antimicrobial agents (polymyxin B and colistin) were tried for fibrous materials modification. Five microliters of 2 g/L antimicrobial agent and 0.3 g/L His_6_-OPH (or 0.5 g/L PGA) complexed with PLE_50_ in a PBS buffer (pH 7.4) or Na-carbonate buffer (pH 9.5) were applied to samples (1 × 1 cm) and dried for 20–22 h at +8 °C under sterile conditions. Then, antibacterial activity was determined, while similar controls were issued.

To investigate the kinetics of cell death, a special series of samples modified by Ta (or Zn) NPs and His_6_-OPH (in complex with PEG-PLE_50_ in Na-carbonate buffer, pH 10.5) was prepared as described above. Bacterial suspension was loaded shortly (i.e., at *t* = 0). After that, samples were withdrawn individually at certain time points of exposure and analyzed.

### 4.5. Hydrolytic Activity of Modified Fibrous Materials

Dried samples of fibrous materials modified by Ta (or Zn) NPs and 1 U of His_6_-OPH (in complex with PEG-PLE_50_ in Na-carbonate buffer, pH 10.5) were placed in a stirred vessel with 1.8 mL of Na-carbonate buffer (pH 10.5). Then, 0.2 mL of 10 mM paraoxon water solution was added, and the kinetics of the product (4-nitrophenol) growth was monitored for 5 min by sampling a 0.1 mL of a reaction medium into 0.9 mL of the same buffer and measuring OD with an Agilent UV-8453 spectrophotometer at 405 nm. After that, a reaction medium was decanted, and samples were gently washed once with 2 mL of the buffer before further use. Similar samples without His_6_-OPH were applied analogously and used as controls. The initial linear parts of kinetic curves (*V*_o_ = tg α) were used to calculate enzymatic activity of modified fiber materials. One unit of enzyme activity was defined as the quantity of the enzyme necessary to hydrolyse 1 µmol of paraoxon per min at 25 °C.

### 4.6. Computational Methods

Crystallographic structure of PvdQ from *Pseudomonas aeruginosa* was obtained from the Protein Data Bank (PDB 4M1J).

The surface charge distribution of enzyme at a certain pH was calculated using the Adaptive Poisson–Boltzmann Solver (APBS) and PDB2PQR servers (ver. 1.4.2.1 and 2.1.1, respectively, available at http://www.poissonboltzmann.org/, accessed on 25 January 2022) with a PARSE force field and default settings [31,32]. Furthermore, the structure was converted from PQR to PDBQT format using AutoDockTools (as part of MGLTools ver. 1.5.6, available at http://mgltools.scripps.edu/, accessed on 25 January 2022) [33]. Polymer ligands (PLE_50_, PLD_50_, PEG-PLE_50_, PEG-PLD_50_, PLE_50_-PEG-PLE_50_) were prepared similarly, as described previously [20].

Simulations of enzyme–ligand complexes were performed at the Supercomputing Center of Lomonosov Moscow State University [34], using up to 512 cores of Intel Xeon X5570 2.93GHz and 1.5 TB of memory. For that, AutoDock Vina (ver. 1.1.2, available at http://vina.scripps.edu/, accessed on 25 January 2022) [35] with Intel MPI Library (ver. 5.0.1) was applied with default program options as published earlier [20]. The grid box was approximately centered on the center of mass of the enzyme and its size was chosen so that enzyme molecule was within the box plus an additional margin. Following the procedure, the “receptor” (i.e., enzyme) was proposed as rigid and the “ligand” (i.e., polymer) was fully flexible. Results were visually inspected for possible errors or molecular intersections, and six poses with minimal energy were selected.

The solvent-accessible area occupied by polymers on the surface of the enzyme was calculated using the “*get_area*” function of PyMOL Molecular Graphics System (ver. 1.7.6, Schrödinger Inc., New York, NY, USA). Statistical analysis was realized using SigmaPlot (ver. 12.5, Systat Software Inc., San Jose, CA, USA) and OriginPro (ver. 9.4.2, OriginLab Corporation, Northampton, MA, USA), and the data are presented as means ± standard deviation (± SD) unless otherwise stated.

## 5. Conclusions

Thus, novel fiber materials of double functionality were successfully obtained in the work via a simple procedure. Up-to-date methods from nanotechnology and biotechnology were combined to produce highly effective materials. The prospectively suggested approach may make it possible to greatly expand the spectrum of the toxic compounds that can be eliminated chemically and/or enzymatically by such protective materials. Namely, the target toxins may be (but not limited to) those produced biologically (like mycotoxins, bacterial toxins, venoms, etc.) and antropogenically (like gases, vapours, solubles, etc.).

## Data Availability

The data presented in this study are available on request from the corresponding author.

## Supplementary Materials

The following are available online at https://www.mdpi.com/article/10.3390/ijms23031359/s1.
