# Supplementary material for: Combined Modification of Fiber Materials by Enzymes and Metal Nanoparticles for Chemical and Biological Protection
Source: Int J Mol Sci. 2022 Jan 25;23(3):1359. doi: 10.3390/ijms23031359 (PMC8835833; doi:10.3390/ijms23031359)
Supplement: Supplementary file 1 [file ijms-23-01359-s001.zip › ijms-1556792-supplementary.pdf]

Supplementary Materials

Combined Modification of Fiber Materials by Enzymes and Metal Nanoparticles for Chemical and Biological Protection

Ilya Lyagin[1,2], Nikolay Stepanov[1,2], George Frolov[3] and Elena Efremenko[1,2,*]

[1] Faculty of Chemistry, Lomonosov Moscow State University, Lenin Hills 1/3, Moscow 119991, Russia; lyagin@mail.ru (I.L.); na.stepanov@gmail.com (N.S.)
[2] N.M. Emanuel Institute of Biochemical Physics RAS, Kosygin str. 4, Moscow 119334, Russia
[3] Department of Physical Chemistry, National University of Science and Technology "MISIS", Leninsky ave. 4, Moscow 119049, Russia; georgifroloff@yandex.ru
* Correspondence: elena_efremenko@list.ru; Tel.:+7-(495)-939-3170; Fax: +7-(495)-939-5417

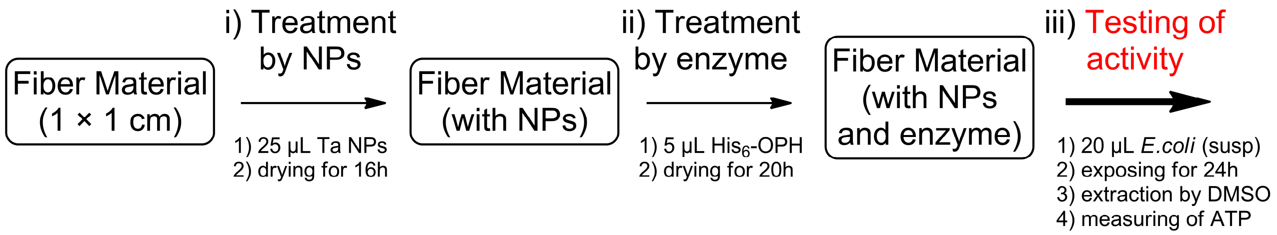

Scheme S1. General work-flow of fiber materials production. As an example, typical conditions for modification by combination of Ta nanoparticles with His_6-OPH were supplied. Activity of ready-to-use fiber materials modified by nanoparticles and enzymes were further analyzed with bacterial cells (*B.subtilis* and *E.coli*) and organophosphorus pesticide (paraoxon).

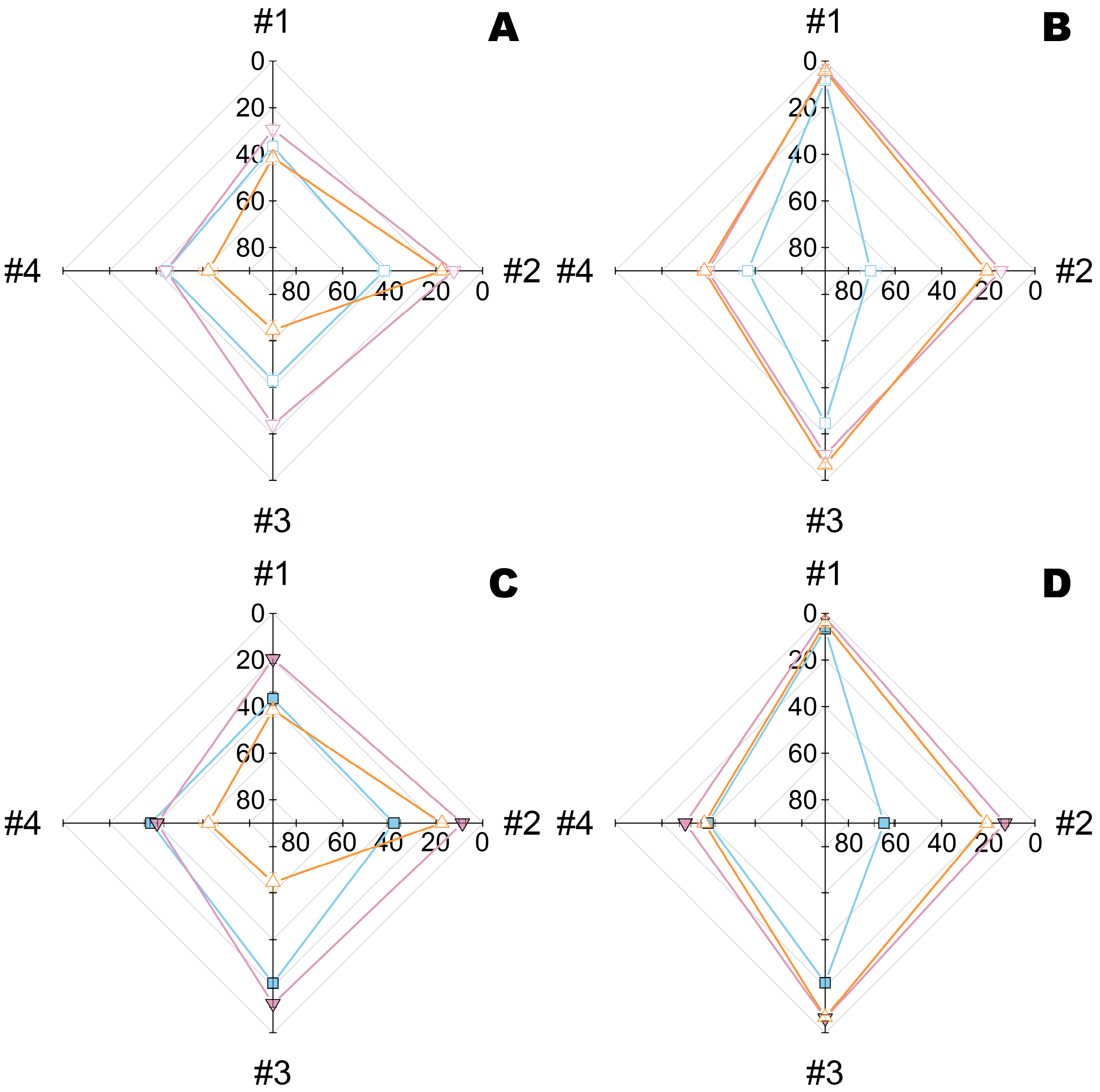

Figure S1. Residual intracellular ATP quantity (%) in *Bacillus subtilis* (**A**, **C**) and *Escherichia coli* bacterial cells (**B**, **D**) exposed for 24 h onto different fiber materials (#1, #2, #3, and #4) treated with Zn (**A**, **B**) or Ta nanoparticles (**C**, **D**), enzyme His6-OPH, or their combinations. ATP of bacterial cells similarly exposed on the same materials without treatment (i.e. controls) was taken as 100%. The His6-OPH was prepared as a complex with PEG-PLE50 in a Na-carbonate buffer (pH 10.5). Designation: □ – Zn nanoparticles, Δ – His6-OPH, ▽ – Zn nanoparticles with His6-OPH, ■ – Ta nanoparticles, ▼ – Ta nanoparticles with His6-OPH.
